# Detection of Porcine Circovirus 1/2/3 and Genetic Analysis of Porcine Circovirus 2 in Wild Boar from Jiangxi Province of China

**DOI:** 10.3390/ani12162021

**Published:** 2022-08-10

**Authors:** Xifeng Hu, Zheng Chen, Yu Li, Zhen Ding, Qinghua Zeng, Tong Wan, Huansheng Wu

**Affiliations:** 1 Department of Preventive Veterinary Medicine, College of Animal Science and Technology, Jiangxi Agricultural University, Zhimin Street, Qingshan Lake, Nanchang 330045, China; 2 Jiangxi Provincial Key Laboratory for Animal Science and Technology, College of Animal Science and Technology, Jiangxi Agricultural University, Nanchang 330045, China; 3 College of Engineering, Jiangxi Agricultural University, Zhimin Street, Qingshan Lake, Nanchang 330045, China

**Keywords:** wild boars, domestic pigs, PCVs, PCV2, PCV3, genotype

## Abstract

**Simple Summary:**

Since PCV2 is currently the primary viral agent in intensive pigs, the cost of immune suppression and preventative measures has resulted in severe economic losses. It has been claimed that PCVs infect wild pigs in a number of nations, including South Korea, Germany, and Italy. Jiangxi province in China is a heavily populated area with an extremely intensive pig production industry, but data on the transmission of PCVs in wild boar are poor. In order to ascertain the incidence of PCV1/2/3 and the genetic characteristics of PCV2 circulating in wild boar in Jiangxi province, an epidemiological investigation was conducted. We discovered a medium prevalence of PCVs, notably PCV2, which indicated that wild boar in Jiangxi province were exposed to PCVs. Our findings highlight the need for the pig sector to actively prevent the contact between livestock and wild boar, which have a high risk of PCVs infection.

**Abstract:**

A number of disorders that harm pig production are linked to porcine circoviruses, including PCV2. PCV2 infection is a substantial contributor to porcine-circovirus-associated illnesses (PCAS) and the post-weaning multi-systemic wasting syndrome (PMWS), which have a significant negative economic impact on pig production. Additionally, PCV infection has been labeled as a global concern to cattle and wildlife. This study’s objectives were to examine the prevalence of PCV1/2/3 in Jiangxi Province, China, and to clarify the epidemiological significance of wild boar in PCV epidemiology. The 2020 hunting seasons resulted in the collection of 138 wild boar samples for PCV1/2/3 detection, which was followed by the genetic clarification of PCV2 strains. According to our data, 21.7% (30/138) of the population had PCV1 positivity, 22.5% (31/138) had PCV2 positivity, and 5.8% (8/138) had PCV3 positivity. Additionally, 10 out of 138 wild boar samples had PCV1 and PCV2 co-infections, while 5 out of 138 wild boar samples had PCV2 and PC3 co-infections. Nineteen full-length PCV2 genomes measuring 1767 nt were recovered from various animal tissues using conventional PCR. Eighteen out of nineteen PCV2 strains were identified as PCV2b by phylogenetic tree analysis, which was completed by the reference strain HLJ2015 obtained from domestic pigs in 2015. Additionally, one genotype of PCV2d JX11-2020 (MW889021) shared a sub-branch with the referenced strain TJ (AY181946), which was isolated in domestic pigs in 2002. This finding raises the possibility that domestic pigs could contract PCV2 strains from wild boar, posing a serious threat to the Jiangxi province of China’s pig production industry.

## 1. Introduction

According to the International Committee on Taxonomy of Viruses, porcine circoviruses (PCV) are categorized as members of the Circoviridae family of viruses [1]. The mature PCV virion has a tiny diameter of less than 20 nm, and is non-enveloped. The PCV genome is circular, single-stranded, and spherical, with a length of roughly 2000 nt [2]. There are currently four distinct PCV genotypes known: PCV1, PCV2, PCV3, and PCV4 [3]. PCV1, a cell-cultured virus with non-pathogenic effects, has been identified in both domestic and wild boar [4,5,6,7]. It is not yet apparent how PCV3 contributes to illness pathogenesis or whether disease-related damage can occur. Additionally, PCV3 has been found in a variety of animals, including wild boar and farmed pigs [8]. In both healthy and diarrheal pigs in 2019, the unique PCV genotype PCV4 was discovered for the first time [9,10,11,12,13]. But it is still unclear exactly how PCV4 causes disease [14]. Contrary to the other PCV genotypes, PCV2 harms domestic and wild pig alike through a number of syndromes such post-weaning multisystemic wasting syndrome (PMWS) and porcine-circovirus-associated disorders (PCV-associated diseases) [15,16,17]. Three main aspects make up the huge PCV2 flood. First, failure to vaccinate pigs with PCV2 leads to immune response suppression, which lowers overall production efficiency [18]. Second, it is extremely expensive to control and prevent PCV2 infection [19]. Third, compared to PCV2 negative pigs, the secondary infection rate was considerably greater in PCV2 positive pigs [20]. According to estimates, PCV2 has a 100 million yuan annual economic impact in China, which indicates that it may have a negative economic impact on intensive pig production [21]. Nine unique PCV2 genotypes have been identified to date (PCV2a, PCV2b, PCV2c, PCV2d, PCV2e, PCV2g, PCV2h, and PCV2i), indicating that the PCV2 genotype circulation has grown increasingly complicated and has likely replaced previously circulating strains with the new genotypes [22,23,24,25,26].

The circular genome of PCV2 and PCV3 is primarily composed of two open reading frames (ORF), with ORF1 encoding the rep protein involved in viral replication and ORF2 expressing the cap protein [20]. Infection with PCV2 or PCV3 is common in nations with a significant swine sector because of the high transmission potential of these viruses [27]. In numerous nations, including Spain, Germany, Italy, and South Korea, wild boar are susceptible to both PCV2 and PCV3 as well as other swine-associated viruses, according to previous literature [28,29,30,31].

Investigations into PCV2 epidemiology in China revealed that domestic pigs were infected with a variety of PCV2 genotypes. Additionally, it has been reported that wild boars in Italy are infected with PCV2 and PCV3, raising the possibility that wild boars may aid in the spread of these viruses to domestic pigs [10]. Additionally, Jiangxi province in China has never disclosed any information on virus transmission, specifically PCV circulation in wild boar. Therefore, 138 different organ samples were taken from wild boars to detect for PCV infection in order to better understand the role of wild boar in PCV transmission in the Jiangxi province. Further research was done on PCV2’s genetic diversity. This study primarily identified the PCV2b genotype of PCV2, which will give the swine sector crucial information to prevent PCV2 infection in the Jiangxi province of China.

## 2. Materials and Methods

### 2.1. Ethical Approval

Since no live animals were used in this study, our study did not require ethical approval. However, to avoid controversy, we still invited the Animal Ethics Committee of Jiangxi Agricultural University to supervise the collection of wild boar samples. All samples were provided by hunters with wild boar hunting licenses according to standard procedures. All wild boar samples were sent to primary processing for purification, and then transported to Jiangxi Provincial Key laboratory for Animal Science and Technology for storage in −80 °C.

### 2.2. Wild Boars Samples Collection Area

**Figure 1:**
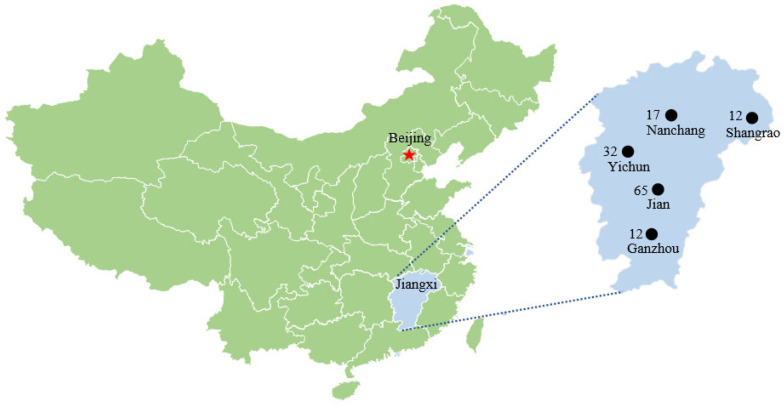
Regions with collected wild boar samples are marked with black dots in Jiangxi province of China.

A total number of 138 samples from different wild boars, including 23 livers, 18 spleens, 35 kidneys, 10 brains, 36 lymphs, and 16 lungs, were collected from five different mountains in Ganzhou, Nanchang, Shangrao, Yichun, and JiAn in Jiangxi province between May and October 2020. In addition, to exclude the virus strain derived from the same positive animals, 138 tissue samples were collected from 138 different wild boars. The details of the number of samples showed in Figure 1 and Table 1.

### 2.3. Viral DNA Extraction

All collected samples were used for detection of porcine circovirus, including PCV1, PCV2, and PCV3, by using real-time PCR assays with Taqman probes. Samples of 25 mg each were used to extract viral DNA. Briefly, samples diluted with 5-fold phosphate-buffered saline (PBS; 0.1 M, pH = 7.4) and were freeze-thawed twice to release virus, then centrifuged at 12,000× *g* for 10 min at 4 °C. The supernatants were separated for the next virus DNA extraction using the TIANamp Virus DNA Isolation Kit (DP315, TIANGEN Biotechnology Co., Ltd., Nanjing, China) according to the manufacturer’s protocols. The extracted DNA was immediately stored until further experimentation.

### 2.4. Differential Detection of PCVs

Using TaqMan-based, real-time quantitative PCR to concurrently test and distinguish between porcine circovirus 1, 2, and 3. The probes from PCV1 and PCV2 were conjugated to FAM and VIC fluorescence signals, respectively, and the probe from PCV3 was conjugated to FAM. The sequence of primers and probes are shown in Table 2, and were synthesized by TSINGKE Biotech Beijing Co., Ltd. (Beijing, China). Each sample was searched with qPCR for the detection of PCV1/PCV2 and PCV3 in two separate wells in a plate. The 20 μL PCR reaction mix contained: 2 μL of viral DNA or control ddH_2_O, 10 μL of 2×T5 Fast qPCR Mix (TSINGKE Biotech Beijing Co., Ltd., Beijing, China), 0.3 μL of each probe (10 μM), and 0.5 μL of each primer (10 μM). The amplification procedure was 95 °C for 30 s, 40 cycles of 95 °C for 5 s, and 60 °C for 1 min. The fluorescence signal was registered at the end of each cycle of the 60 °C extension step. The cycle threshold (Ct) was determined based on the protocol phase of each reaction. A Ct value of less than 37 was positive.

### 2.5. Full Genome of PCV2 Amplification and Sequencing

Since PCV1 is non-pathogenic for pigs and the *Ct* value of PCV3 is too high with a weak positive result, we attempted to amplify the full length of PCV2 in this study. In order to obtain the complete genome sequence of PCV2, a pair of primers were first designed based on the referenced sequence (accession number: KY126314.1). The PCR protocol was performed in a 50 μL reaction volume: 25 μL 2× Rapid Taq Master Mix (Green Dye Plus, P222-01, Vazyme Biotechnology Co., Ltd., Nanjing, China), 3 μL template DNA, 1 μL each primer (10 mM), and 17 μL ddH_2_O up to a total of 50 μL. The PCR program was given as follows: pre-denaturation at 95 °C for 5 min and then amplified by 35 cycles of 95 °C for 30 s, annealing at 56 °C for 30 s and stretch at 72 °C for 90 s and a final stretch at 72 °C for 10 min. The purified PCR production was sub-cloned into pMD18T (TAKARA, Takara Biomedical Technology (Beijing) Co., Ltd. Beijing, China) and the positive clone was directly obtained from and sequenced by TSINGKE Biotech Beijing Co., Ltd.

### 2.6. Wild Boars PCV2 Sequence Alignment and Phylogenetic Analysis

In this study, 19 complete genomes of wild boar-derived PCV2 strains were successfully sequenced (Table 3). The complete genome sequences of PCV2 were edited and assembled using DNAstar software (DNAStar V7.1, Madison, WI, USA). MEGA 7.0 software was used to match PCV2 and reference strains entered into GenBank. Phylogenetic trees were also constructed using the neighbor joining method with bootstrap analysis of 1000 replicates in MEGA 7.0 software.

## 3. Results

### 3.1. Prevalence of PCVs in Wild Boars

All one hundred thirty-eight clinical samples from wild boars were examined for PCV1–PCV3 using a TaqMan-based qPCR test. According to findings in Table 4, the infection rates for PCV1, PCV2, and PCV3 were 30/138 (21.73%), 31/138 (22.46%), and 8/138 (5.79%), respectively. Ten samples had PCV1 and PCV2 co-infections (7.25%), while five samples had PCV2 and PC3 co-infections (3.62%), indicating that wild boar in Jiangxi province may have several PCVs co-infected.

We then looked at the PCV detection data in the various organs. According to findings in Table 5, 30 PCV1 positive specimens contained 16 lymph nodes, 7 lung nodes, 5 kidney nodes, and 2 spleen nodes; 31 PCV2 positive samples were 20 lymph nodes, 7 kidney nodes, 3 spleen nodes, and 1 lung; and 8 PCV3 positive specimens contained 5 lymph nodes and 3 kidney nodes. In the liver and brain tissue samples that were collected, we were unable to find any PCV.

### 3.2. Amplification of PCV2 Complete Genome

**Figure 2:**
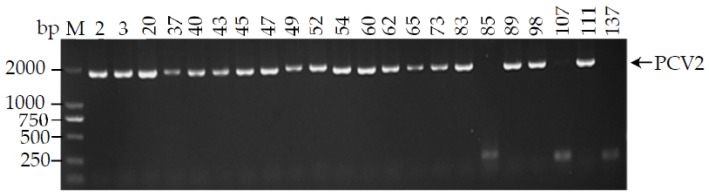
Partial PCV2 positive samples were used to amplify the complete genome of PCV2. The PCR products with about 2000 bp were separately by 1% agarose gel electrophoresis. M: DL2000 marker.

We tried to amplify the entire length of PCV2 in this investigation because PCV1 does not harm pigs and the *Ct* value of PCV3 was higher than 32. The samples (*n* = 22) with a *Ct* value less than 30 were chosen to amplify the PCV2 genome using conventional PCR in order to increase the amplification efficiency. A total of 19 out of 22 wild boar samples were effective in amplifying the PCV2 gene, as seen in Figure 2. The 19 PCV2 genomic segments were subsequently introduced into pMD18-T for further sequencing. The 1767 bp length sequences were all deposited in the NCBI gene repository. As indicated in Table 3, we obtained accession numbers MW889012–MW889030 for PCV2 strains JX2-2020–JX20–2020.

### 3.3. Genetic Analysis of PCV2

**Figure 3:**
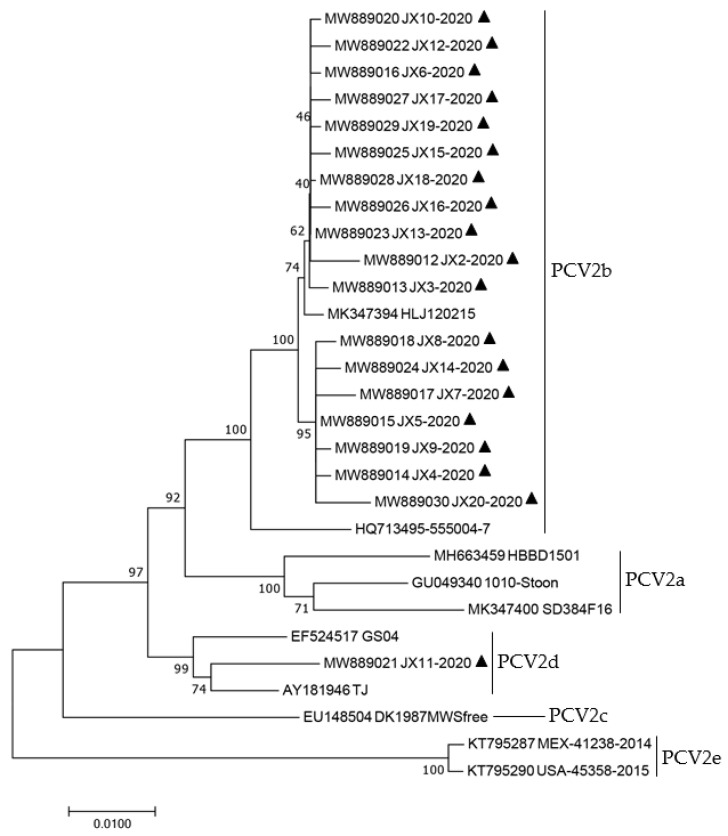
Phylogenetic tree based on the complete genome of all sequences (19 PCV2 sequences amplified in this study and 10 PCV2 reference sequences). The sequences obtained in this study were marked with black triangle. Phylogenetic tree was conducted by using the p-distance-based neighbor-joining (NJ) method with bootstrap analysis of 1000 replicates in MEGA 7.0 software.

**Figure 4:**
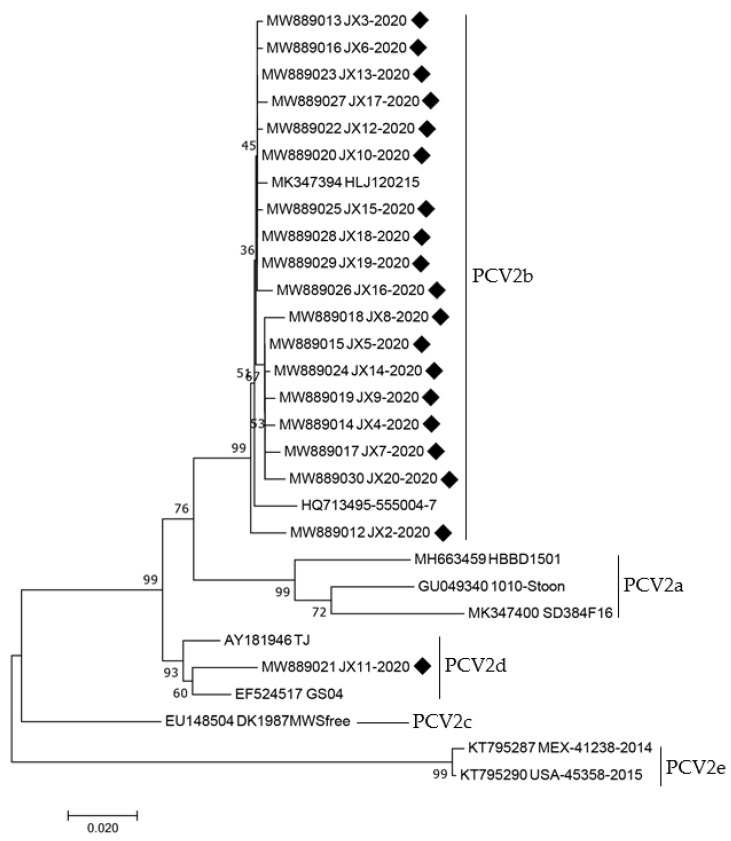
Phylogenetic tree based on the ORF2 amino acids of 19 PCV2 sequences amplified in this study and 10 PCV2 reference sequences). The sequences obtained in this study have been marked with a black square. The phylogenetic tree was performed using the p-distance-based neighbor joining (NJ) method with bootstrap analysis of 1000 replicates in MEGA 7.0 software.

The genomes of all 19 strains were used to construct a phylogenetic tree, which also included 10 reference strains with genotypes (PCV2a–e) (Table 6). According to the findings, only one PCV2 strain belonged to PCV2d while 18 PCV2 strains belonged to PCV2b (Figure 3). The one genotype PCV2d JX11-2020 (MW889021) was in the same sub-branch as the cited strain TJ (AY181946), which was isolated in domestic pigs in 2002. The 18 genotype PCV2b strains were closed for the referenced strain HLJ20215 (MK347394), originating from domestic pigs isolated in 2015. A phylogenetic tree based on the ORF2 amino acids was further built to analyze the properties of PCV2 strains produced from wild boar in this study (Figure 4)**.** A similar outcome to that of the entire genome based on the phylogenetic tree was seen in the phylogenetic tree based on the ORF2 amino acids, as shown in Figure 3. These findings reveal that PCV2-infected feral pigs are a significant potential danger to the pig production business in China since PCV2 strains recovered from feral pigs in this investigation were remarkably similar to PCV2 strains obtained from domestic pigs. Thus, limiting the population of feral pigs and avoiding direct contact between wild boar and domestic pigs should aid in limiting the spread of PCV2 among intensive pigs, thereby minimizing the financial loss of pig farms.

## 4. Discussion

This study looked at PCV1, PCV2, and PCV3 circulation in the Jiangxi wild boar population in China. The PCV infection in wild boar from Jiangxi province has never been described before. A number of studies have examined the prevalence of PCV2, PCV3, or PCV4 in China and shown that domestic pigs are carriers of PCVs [3]. Wild boar samples were gathered for the detection of medium prevalence PCV1/2/3. Our results, when compared to earlier research, revealed that the prevalence of PCV2 or PCV3 infection was far lower than in European nations like Italy or Sardinia [32]. The primary cause of PCVAD is PCV2. Numerous investigations have demonstrated that PCV2 was frequently clinically co-infected with other PCVs, such as PCV3 or PCV4, in domestic pigs [33,34]. Although 138 tissue samples from 138 animals with various organs were collected, this may not accurately represent the PCV infection rate. Our findings showed that lymph nodes had a greater rate of PCV infection detection than other organs. Previous research demonstrated that PCV infection seriously impairs the host’s immune system, including the lymphatic and renal systems. Additionally, finding PCV infection in discarded samples demonstrated that many domestic pig organs might be found to be contaminated with PCV [35,36].

The PCV2a genotype was initially discovered in Chinese pig herds. The PCV2a to PCV2d genotype switch occurred in 2003 [37]. Recently, PCV2d has emerged as the predominant genotype circulating in Chinese domestic pigs [38]. Following genetic analysis, we discovered that one PCV2 strain and 18 PCV2-infected strains belonged to the PCV2b and PCV2d genotypes, respectively. We are unable to amplify the PCV3 genome. Further research will be necessary to determine the prevalence and genotypes of PCV3 infection in wild boar. Identification of PCV2 strains with PCV2b and PCV2d genotypes using phylogenetic analysis. The frequency of PCV in wild boar in Jiangxi province as well as the genotypes of PCV2b and PCV2d in wild boar are thus described in this paper. Since the cap gene of PCV2 in this study’s cited two PCV2 strains from China was relatively stable, it is possible that the genotype of PCV2 infection in wild boars is related to that in domestic pigs. In addition, previous literature discovered that PCV2 strains could be transmitted between wild boars and domestic pigs in Italy [39]. MK347394 reference strain was taken from Heilongjiang province, which is although geographical distant from Jiangxi province. These results suggested that PCV2b might spread more widely between wild boars and domestic pigs. However, whether PCV2d will become the predominant strains in wild boars required further investigations. In addition, the more complex PCV2 genotypes in wild boar need to be studied next, since novel genotypes have so far been gradually discovered in farmed pigs in China.

Wild boar population is uncontrolled and can move freely. The wild boars to carry the new genotype PCV2d were first found in China. However, there has been no confirmed instance of wild boar to domestic pig PCV2d genotype transmission in China. Both pig agriculture and veterinarian public health may still be at risk from this possible spread. We are all aware that PCAD, or swine circovirus associated diseases, have been shown to significantly harm the swine production industry. China has the world’s largest pig producing business, which increases the economic cost of stopping PCV2 circulation there.

This study did have certain limits, though. First, because PCV2’s pathogenicity in wild boars is currently unclear, the samples were randomly collected, and the results showing PCV2 positivity cannot reflect PCV2’s pathogenicity. Second, the small number of samples from Jiangxi province limited the prevalence of PCVs in wild boars. Third, we are unable to collect wild boar PCV3 genotyping data. Therefore, additional extensive wild boar sample studies must be conducted in subsequent research.

## 5. Conclusions

Overall, this study demonstrates widespread PCV circulation, especially PCV2 in Jinagxi province’s wild boar population. According to our research, wild boars are very susceptible to contracting PCV2b and PCV2d, which poses a serious risk to domestic pigs. Since widespread PCV2 infection can substantially impair economic growth in the pig production business, the control of wild boar infected with PCV2 is vital for both the intensive pig farming industry and for public health in veterinary medicine.

## Figures and Tables

**Table 1:** Total number of harvesting wild boar samples in Jiangxi province.

Shangrao	JiAn	Yichun	Ganzhou	Nanchang	Total
12	65	32	12	17	138

**Table 2:** Primers and probes the TaqMan detection assay used in this study.

Assays	Primers /Probes	Sequences (5′-3′)	Genes	Amplicons
Duplex real-time PCR	PCV1F	AACCCCATAAGAGGTGGGTGTT	ORF1	129 bp
	PCV1R	TTCTACCCTCTTCCAAACCTTCCT		
	PCV1-P	VIC-TCCGAGGAGGAGAAAAACAA AATACGGGA-BHQ1		
	PCV2F	CTGAGTCTTTTTTATCACTTCGTAATGGT	ORF1-ORF2	146 bp
	PCV2R	ACTGCGTTCGAAAACAGTATATACGA		
	PCV2-P	FAM-TTAAGTGGGGGGTCTTTAAGATTA AATTC TCTGAATTGT-BHQ1		
Real-time PCR	PCV3F	ACTGCGTTCGAAAACAGTATATACGA	ORF2	111 bp
	PCV3R	CATAAATGCTCCAAAGCAGTGCT		
	PCV3-P	VIC-ATATGTGTTGAGCCATGGGGTGGG TCT-BHQ1		
Traditional PCR	PCV2FF	TAATAAAAACCATTACGAAGTGATA	Full genome	2000 bp
	PCV2FR	GGTTTTTATTATTCATTAAGGGTTA		

**Table 3:** The information of PCV2 strains obtained in this study.

No.	Accession Number	Strains	Country	Year	Host	Length	Genotype	Organs
1	MW889012	JX2-2020	China	2020	Wild boars	1767 bp	PCV2b	lymph node
2	MW889013	JX3-2020	China	2020	Wild boars	1767 bp	PCV2b	lymph node
3	MW889014	JX4-2020	China	2020	Wild boars	1767 bp	PCV2b	kidney
4	MW889015	JX5-2020	China	2020	Wild boars	1767 bp	PCV2b	lymph node
5	MW889016	JX6-2020	China	2020	Wild boars	1767 bp	PCV2b	lymph node
6	MW889017	JX7-2020	China	2020	Wild boars	1767 bp	PCV2b	spleen
7	MW889018	JX8-2020	China	2020	Wild boars	1767 bp	PCV2b	lymph node
8	MW889019	JX9-2020	China	2020	Wild boars	1767 bp	PCV2b	lymph node
9	MW889020	JX10-2020	China	2020	Wild boars	1767 bp	PCV2b	lymph node
10	MW889021	JX11-2020	China	2020	Wild boars	1767 bp	PCV2b	lymph node
11	MW889022	JX12-2020	China	2020	Wild boars	1767 bp	PCV2b	lymph node
12	MW889023	JX13-2020	China	2020	Wild boars	1767 bp	PCV2b	lymph node
13	MW889024	JX14-2020	China	2020	Wild boars	1767 bp	PCV2b	lymph node
14	MW889025	JX15-2020	China	2020	Wild boars	1767 bp	PCV2d	lymph node
15	MW889026	JX16-2020	China	2020	Wild boars	1767 bp	PCV2b	lymph node
16	MW889027	JX17-2020	China	2020	Wild boars	1767 bp	PCV2b	lymph node
17	MW889028	JX18-2020	China	2020	Wild boars	1767 bp	PCV2b	kidney
18	MW889029	JX19-2020	China	2020	Wild boars	1767 bp	PCV2b	kidney
19	MW889030	JX20-2020	China	2020	Wild boars	1767 bp	PCV2b	kidney

**Table 4:** Summary of PCVs detection in 138 wild boar samples.

	Porcine Circovirus Genotype				
	PCV1	PCV2	PCV3	PCV1 + PCV2	PCV2 + PCV3
Positive (*n*)	30	31	8	10	5
Total (*n*)	138	138	138	138	138
Rate (%)	21.73	22.46	5.79	7.25	3.62

**Table 5:** Detection results of PCVs in different organs of wild boars.

No.	Organ	1	2	3	No.	Organ	1	2	3	No.	Organ	1	2	3
1	liver	−	−	−	48	lymph node	+	−	−	94	lung	+	−	−
2	kidney	−	+	+	49	kidney	−	+	−	95	kidney	−	−	−
3	spleen	−	+	−	50	lymph node	−	−	−	96	lung	−	−	−
4	brain	−	−	−	51	liver	−	−	−	97	kidney	−	−	−
5	lymph node	+	−	−	52	kidney	−	+	−	98	kidney	+	+	−
6	kidney	−	−	−	53	Liver	−	−	−	99	lung	−	−	−
7	kidney	−	−	−	54	lymph node	−	+	−	100	kidney	−	−	−
8	lung	+	−	−	55	kidney	−	−	−	101	brain	−	−	−
9	lung	−	−	−	56	liver	−	−	−	102	kidney	−	−	−
10	lung	+	−	−	57	liver	−	−	−	103	lung	+	−	−
11	spleen	−	−	−	58	brain	−	−	−	104	kidney	−	−	−
12	spleen	+	−	−	59	spleen	−	−	−	105	lymph node	+	−	−
13	spleen	−	−	−	60	lymph node	−	+	−	106	lung	−	−	−
14	kidney	−	−	−	61	spleen	−	−	−	107	lymph node	−	+	+
15	kidney	−	−	−	62	lymph node	+	+	−	108	lymph node	+	+	−
17	brain	−	−	−	63	spleen	−	−	−	109	lung	−	−	−
18	brain	−	−	−	64	kidney	−	−	−	110	lymph node	−	−	−
19	kidney	−	−	−	65	kidney	−	+	−	111	spleen	−	+	−
20	lymph node	−	+	−	66	brain	−	−	−	112	spleen node	−	−	−
21	lymph node	−	−	−	67	liver	−	−	−	113	lymph node	−	+	−
22	lymph node	−	−	−	68	lymph node	−	−	+	114	lymph node	−	−	−
23	kidney	+	−	−	69	lymph node	−	−	+	115	lung	−	−	−
24	kidney	−	−	−	70	liver	−	−	−	116	lymph node	+	−	−
25	spleen	−	−	−	71	kidney	−	−	−	117	spleen	−	−	−
26	brain	−	−	−	72	kidney	−	−	−	118	lung	−	−	−
27	spleen	+	−	−	73	lymph node	+	+	−	119	kidney	+	−	−
28	kidney	+	−	−	74	liver	−	−	−	120	spleen	+	−	−
29	spleen	−	−	−	75	kidney	−	−	+	121	liver	−	−	−
30	kidney	−	−	−	76	liver	−	−	−	122	brain	−	−	−
31	liver	−	−	−	77	lung	−	−	−	123	lymph node	−	+	−
32	liver	−	−	−	78	brain	−	−	−	124	liver	−	−	−
33	spleen	−	−	−	79	liver	−	−	−	125	lymph node	−	+	−
34	lymph node	+	−	−	80	kidney	−	−	−	126	liver	−	−	−
35	liver	−	−	−	81	kidney	+	−	−	127	liver	−	−	−
36	kidney	−	−	−	82	kidney	−	−	−	128	lymph node	−	+	−
37	lymph node	−	+		83	lymph node	+	+	−	129	liver	−	−	−
38	lymph node	+	−	−	84	lung	−	−	−	130	spleen	−	−	−
39	kidney	−	−	−	85	lymph node	+	+	−	131	liver	−	−	−
40	lymph node	+	+		86	lung	−	−	−	132	spleen	−	+	−
41	lymph node	+	−	−	87	lymph node	−	−	−	133	lymph node	−	−	−
42	kidney	−	−	−	88	lung	+	−	−	134	lymph node	−	+	+
43	lymph node	+	+	−	89	lymph node	+	+	−	135	liver	−	−	−
44	kidney	−	−	−	90	liver	−	−	−	136	spleen	−	−	−
45	lymph node	−	+	+	91	liver	−	−	−	137	kidney	−	+	−
46	kidney	−	−	−	92	kidney	−	+	+	138	liver	−	−	−
47	lymph node	+	+	−	93	lung	+	+	−					

1: PCV1; 2: PCV2; 3: PCV3.

**Table 6:** The information of 22 referenced PCV2 strains.

No.	Accession Number	Strains	Genotype	Country	Year	Host
1	GU049340	1010-Stoon	PCV2a	Cannada	2007	wild boar
2	MH663459	HBBD1501	PCV2a	China	2015	domestic pig
3	MK347400	SD384F16	PCV2a	China	2016	domestic pig
4	HQ713495	5-55004-7	PCV2b	USA	2005	domestic pig
5	MK347394	HLJ120215	PCV2b	China	2015	domestic pig
6	EU148504	DK1987PMWSfree	PCV2c	Denmark	2007	domestic pig
7	EF524517	GS04	PCV2d	China	2007	wild boar
8	AY181946	TJ	PCV2d	China	2002	domestic pig
9	KT795287	MEX-41238-2014	PCV2e	Mexico	2014	wild boar
10	KT795290	USA-45358-2015	PCV2e	USA	2015	wild boar

## Data Availability

All the data analyzed in this study are available from corresponding author.
